# Rethinking the combination treatment of fulvestrant and anastrozole for metastatic breast cancer: an integrated reanalysis of aromatase–estrogen receptor axis

**DOI:** 10.1186/s40169-019-0246-5

**Published:** 2019-11-18

**Authors:** Xing Huang

**Affiliations:** 1 Zhejiang Provincial Key Laboratory of Pancreatic Disease, The First Affiliated Hospital, School of Medicine, Zhejiang University, 79 Qingchun Road, Hangzhou, 310003 Zhejiang China; 2 The Key Laboratory of Developmental Genes and Human Disease, Institute of Life Sciences, Southeast University, Nanjing, 210096 Jiangsu China

**Keywords:** Anastrozole, Aromatase, Fulvestrant, Estrogen receptor, Combination therapy, Prognostic analysis, Immune relevance

## Abstract

Aberrant expression or hyperactivation of aromatase (CYP19A1)–estrogen receptor (ESR) axis is well identified as one of the major causes of breast cancer. Lots of drugs have been developed for targeting CYP19A1 or ESR respectively, such as anastrozole and fulvestrant. Recently, Mehta et al. reported in *NEJM* that the combined treatment of anastrozole and fulvestrant increased long-term survival of patients with metastatic breast cancer, especially for those without receiving endocrine therapy. However, the integrated prognostic analyses of CYP19A1 and ESR1/ESR2 indicated some contradictory outcomes to the recent clinical trial. Moreover, immunological investigation further revealed that targeting the whole CYP19A1–ESR axis might cause the inactivation of anti-tumor immune response, which largely attenuated its application prospects in breast cancer. Considered the pathophysiologic functions of CYP19A1 and ESR1/ESR2-mediated signaling pathway in breast cancer seem as more complicated than what we have already known, more precise evaluation will be needed in urgent.

## Background

Aberrant expression or hyperactivation of aromatase (CYP19A1)–estrogen receptor (ESR) axis is well identified as one of the major causes of breast cancer. Lots of drugs have been developed for targeting CYP19A1 or ESR respectively, such as anastrozole and fulvestrant. Recently, I read with great interest and respect the clinical study in *NJEM* from Mehta et al. [1], reporting the combined treatment of anastrozole and fulvestrant increases long-term survival of patients with metastatic breast cancer, especially for those without receiving endocrine therapy [2, 3]. Indeed, the outcome is intriguing, but it still warrants further discussion.

## Main text

**Fig. 1:**
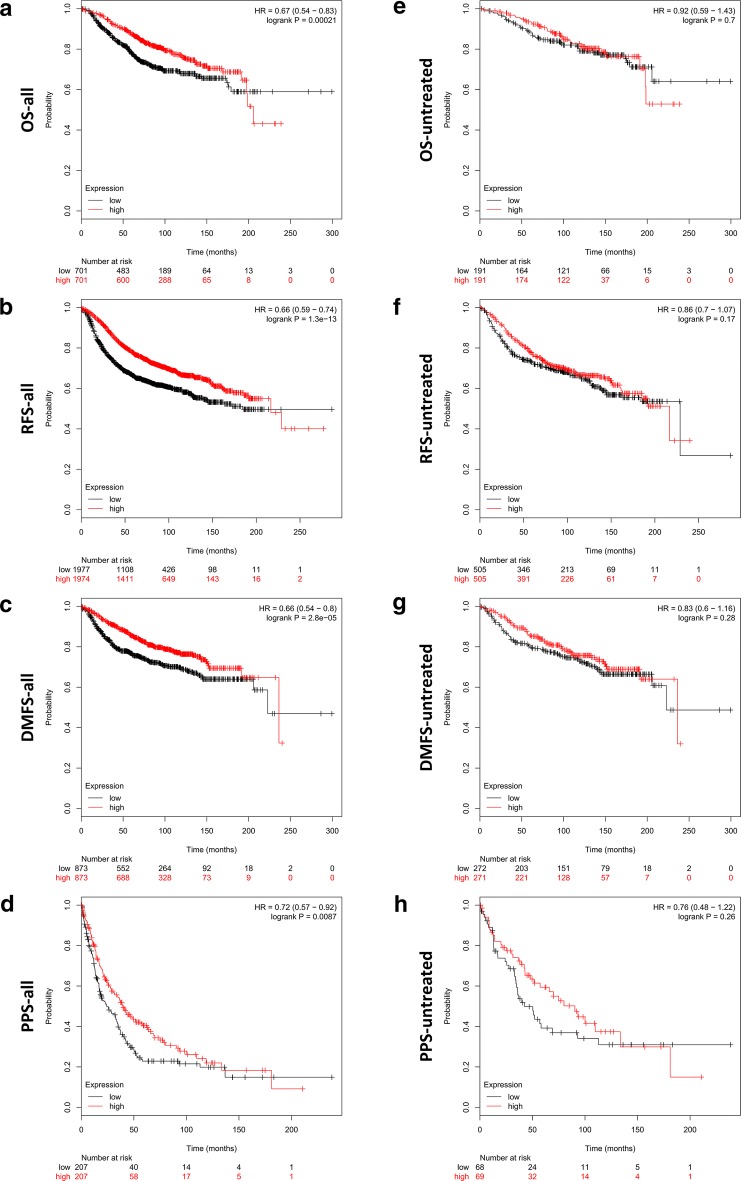
Prognostic analyses of CYP19A1–ESR axis in breast cancer. **a**–**d** OS, RFS, DMFS and PPS of integrated CYP19A1 and ESR1/ESR2 in breast cancer patients (n = 1402 in OS, 3951 in RFS, 1746 in DMFS, 414 in PPS respectively). **e**–**h** OS, RFS, DMFS and PPS of integrated CYP19A1 and ESR1/ESR2 in untreated breast cancer patients (n = 382 in OS, 1010 in RFS, 543 in DMFS, 137 in PPS respectively). The detailed HR and logrank p-value were individually shown as indicated in each panel, and p-value < 0.05 was considered statistically significant

Aromatase (CYP19A1)–estrogen receptor (ESR) axis is deemed as the synergistic target for the combination of anastrozole and fulvestrant [4, 5]. However, the integrated prognostic analyses of CYP19A1 and ESR1/ESR2 showed contradictory outcomes to the recent clinical trial. Briefly, the high expression levels of CYP19A1 and ESR1/ESR2 significantly favored (rather than supposedly un-favored) the overall survival (OS) (HR, 0.67; 95% CI 0.54 to 0.83; *p* < 0.001) (Fig. 1a), relapse free survival (RFS) (HR, 0.66; 95% CI 0.59 to 0.74; *p* < 0.001) (Fig. 1b), distant metastasis free survival (DMFS) (HR, 0.66; 95% CI 0.54 to 0.8; *p* < 0.001) (Fig. 1c), as well as post progression survival (PPS) (HR, 0.72; 95% CI 0.57 to 0.92; *p* < 0.001) (Fig. 1d) of breast cancer patients (all cases). Moreover, in contrast to the previous report, the patients without receiving relevant therapies (untreated cases) showed non-significant correlation between CYP19A1–ESR axis and OS (HR, 0.92; 95% CI 0.59 to 1.43; *p* = 0.7) (Fig. 1e), RFS (HR, 0.86; 95% CI 0.7 to 1.07; *p* = 0.17) (Fig. 1f), DMFS (HR, 0.83; 95% CI 0.6 to 1.16; *p* = 0.28) (Fig. 1g), and PPS (HR, 0.76; 95% CI 0.48 to 1.22; *p* = 0.26) (Fig. 1h). Obviously, the clinical discordance in outcomes from therapeutic trial and genomic analyses could not be well explained by the present knowledge to CYP19A1–ESR axis. Hence, it would be interesting in future research to elucidate whether the previous benefit from anastrozole–fulvestrant combination is actually caused by artificial effects of combination therapy [6, 7].

**Fig. 2:**
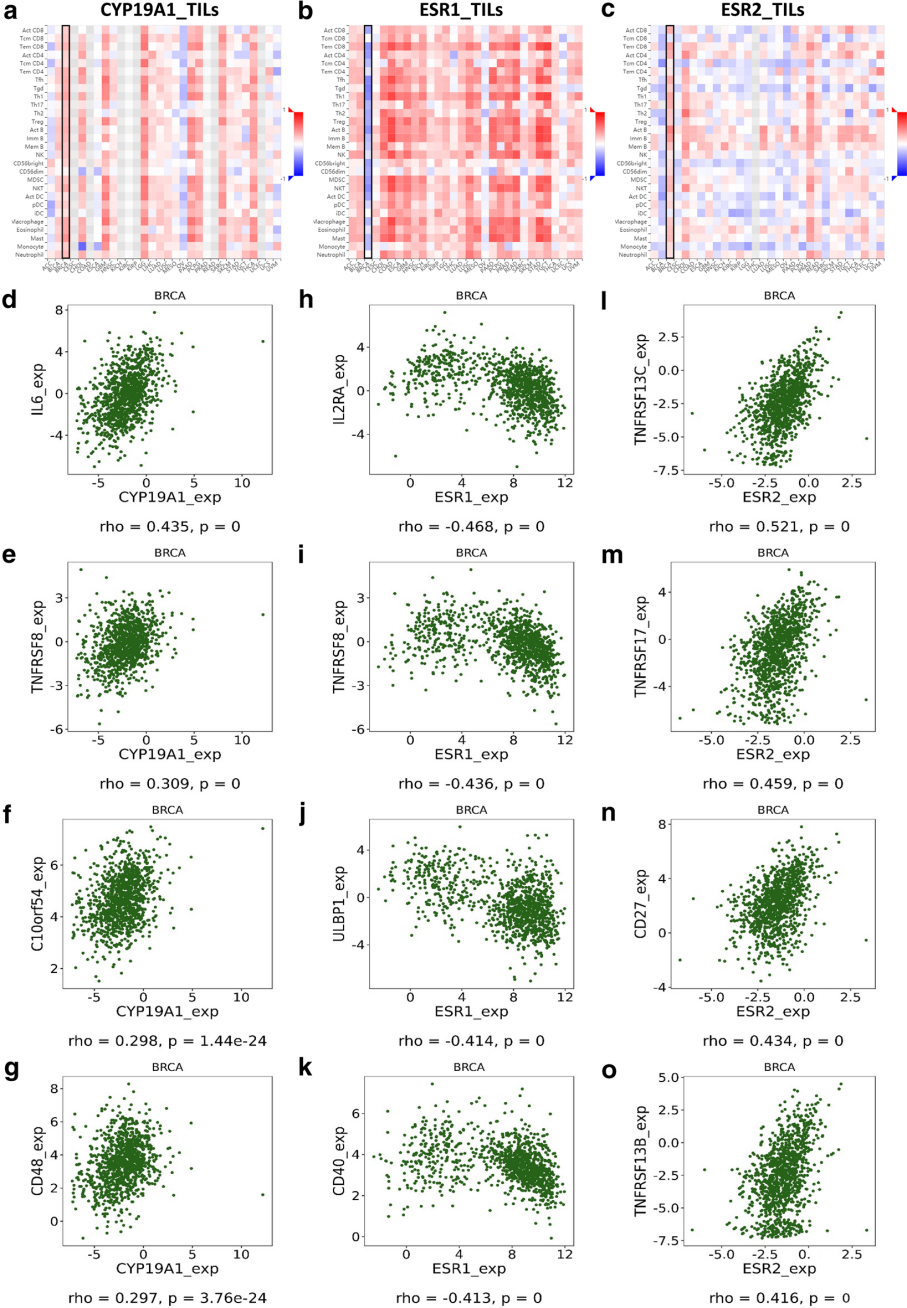
Immunological analyses of CYP19A1–ESR axis in breast cancer. **a**–**c** Spearman correlations between CYP19A1, ESR1, ESR2 and multiple TILs across human cancers. For each cancer type, the relative abundance of TILs were inferred by using GSVA based on gene expression profile (TCGA). The readouts of BRCA were highlighted in black frames. **d**–**g** Spearman correlations between CYP19A1 and representative immunostimulators (including IL6, TNFRSF8, C10orf54 and CD48) in BRCA (TCGA). **h**–**k** Spearman correlations between ESR1 and representative immunostimulators (including IL2RA, TNFRSF8, ULBP1 and CD40) in BRCA (TCGA). **l**–**o** Spearman correlations between ESR2 and representative immunostimulators (including TNFRSF13C, TNFRSF17, CD27 and TNFRSF13B) in BRCA (TCGA). The detailed rho and p-value were individually shown as indicated in each panel, and p-value < 0.05 was considered statistically significant

Intriguingly, several studies had indicated the potential connection between CYP19A1–ESR axis and immune system [8–10]. To better understand the regulatory effects of CYP19A1 and ESR1/ESR2 on breast cancer, immunological analyses were performed to demonstrate the detailed relationships between CYP19A1, ESR1, ESR2 and breast cancer immunity. Surprisingly, it was observed that the expression level of CYP19A1 correlated positively with the relative abundance of tumor-infiltrating lymphocytes (TILs) in breast cancer (Fig. 2a). By contrast, ESR1 was observed negatively related to most TILs (Fig. 2b); whereas similar to CYP19A1 but not ESR1, ESR2 was found correlated positively with TILs (Fig. 2c). Moreover, further analyses showed that CYP19A1 positively related to many key immunostimulators, including but not limited to, interleukin 6 (IL6, rho = 0.435) (Fig. 2d), lymphocyte activation antigen CD30 (TNFRSF8, rho = 0.309) (Fig. 2e), V-type immunoglobulin domain-containing suppressor of T cell activation (VISTA/C10orf54, rho = 0.298) (Fig. 2f), and signaling lymphocytic activation molecule family 2 (SLAMF2/CD48, rho = 0.297) (Fig. 2g). In contrast to CYP19A1, ESR1 correlated negatively with a few of critical immunostimulators, such as interleukin 2 receptor subunit alpha (IL2RA, rho = − 0.468) (Fig. 2h), TNFRSF8 (rho = − 0.436) (Fig. 2i), NKG2D ligand 1 (NKG2DL1/ULBP1, rho = − 0.414) (Fig. 2j), and B cell surface antigen CD40 (rho = − 0.413) (Fig. 2k). Accordingly, ESR2 was observed positively related to some important immunostimulators, like B cell-activating factor receptor (CD268/TNFRSF13C, rho = 0.521) (Fig. 2l), B cell maturation factor (CD269/TNFRSF17, rho = 0.459) (Fig. 2m), T cell activation antigen S152 (CD27, rho = 0.434) (Fig. 2n), and transmembrane activator and CAML interactor (CD267/TNFRSF13B, rho = 0.416) (Fig. 2o). These observation strongly suggested that even for conventional targeted therapy for breast cancer, the concomitant immunological impacts should not be neglected, especially in clinical evaluation.

## Conclusions

Taken together, the genomic and immunologic analyses do not support the combined therapeutic strategy of anastrozole and fulvestrant. Although sometimes the outcomes are good, targeting the whole CYP19A1–ESR axis may cause the inactivation of anti-tumor immune response, which largely attenuates its application prospects in breast cancer. Considered the pathophysiologic functions of CYP19A1 and ESR1/ESR2-mediated signaling pathway in breast cancer seem as more complicated than what we have already known or identified, more precise therapy will be needed in the near future.

## Data Availability

All data generated or analyzed during this study are included in this published article.

## Abbreviations

CYP19A1: aromatase
ESR: estrogen receptor
OS: overall survival
RFS: relapse free survival
DMFS: distant metastasis free survival
PPS: post progression survival
TILs: tumor-infiltrating lymphocytes
IL6: interleukin 6
TNFRSF8: lymphocyte activation antigen CD30
C10orf54: V-type immunoglobulin domain-containing suppressor of T cell activation
CD48: signaling lymphocytic activation molecule family 2
IL2RA: interleukin 2 receptor subunit alpha
ULBP1: NKG2D ligand 1
TNFRSF13C: B cell-activating factor receptor
TNFRSF17: B cell maturation factor
CD27: T cell activation antigen S152
TNFRSF13B: transmembrane activator and CAML interactor
